# 3D Printing of Personalized Thick and Perfusable Cardiac Patches and Hearts

**DOI:** 10.1002/advs.201900344

**Published:** 2019-04-15

**Authors:** Nadav Noor, Assaf Shapira, Reuven Edri, Idan Gal, Lior Wertheim, Tal Dvir

**Affiliations:** ^1^ The School for Molecular Cell Biology and Biotechnology Faculty of Life Sciences Tel Aviv University Tel Aviv 6997801 Israel; ^2^ Department of Materials Science and Engineering Faculty of Engineering Tel Aviv University Tel Aviv 6997801 Israel; ^3^ The Center for Nanoscience and Nanotechnology Tel Aviv University Tel Aviv 6997801 Israel; ^4^ Sagol Center for Regenerative Biotechnology Tel Aviv University Tel Aviv 6997801 Israel

**Keywords:** 3D printing, hearts, hydrogels, induced pluripotent stem cells (iPSCs), tissue and organ engineering

## Abstract

Generation of thick vascularized tissues that fully match the patient still remains an unmet challenge in cardiac tissue engineering. Here, a simple approach to 3D‐print thick, vascularized, and perfusable cardiac patches that completely match the immunological, cellular, biochemical, and anatomical properties of the patient is reported. To this end, a biopsy of an omental tissue is taken from patients. While the cells are reprogrammed to become pluripotent stem cells, and differentiated to cardiomyocytes and endothelial cells, the extracellular matrix is processed into a personalized hydrogel. Following, the two cell types are separately combined with hydrogels to form bioinks for the parenchymal cardiac tissue and blood vessels. The ability to print functional vascularized patches according to the patient's anatomy is demonstrated. Blood vessel architecture is further improved by mathematical modeling of oxygen transfer. The structure and function of the patches are studied in vitro, and cardiac cell morphology is assessed after transplantation, revealing elongated cardiomyocytes with massive actinin striation. Finally, as a proof of concept, cellularized human hearts with a natural architecture are printed. These results demonstrate the potential of the approach for engineering personalized tissues and organs, or for drug screening in an appropriate anatomical structure and patient‐specific biochemical microenvironment.

## 1 Introduction

Cardiovascular diseases are the number one cause of death in industrialized nations.^1^ To date, heart transplantation is the only treatment for patients with end‐stage heart failure. Since the number of cardiac donors is limited, there is a need to develop new approaches to regenerate the infarcted heart.^2^ Cardiac tissue engineering provides an alternative approach by integrating cardiac cells and 3D biomaterials.^3^, ^4^ The latter serve as temporary scaffolds, mechanically supporting the cells and promoting their reorganization into a functional tissue.^5^ Following in vitro maturation, the engineered cardiac patch can be transplanted onto the defected heart. When full integration to the host commences, the biomaterials gradually degrade, leaving a functional living patch that regenerates the heart.^3^

The biocompatibility of the scaffolding materials is a crucial factor for eliminating the risk of implant rejection, which jeopardizes the success of the treatment. Therefore, the material itself or its degradation products should be carefully selected.^6^ Most ideally, the biomaterial should possess biochemical, mechanical, and topographical properties similar to those of native tissues. Decellularized tissue‐based scaffolds from different sources meet most of these requirements.^7^, ^8^ However, to ensure minimal response of the immune system, completely autologous materials are preferred.^9^

Recently, our group has shown a new concept for engineering fully personalized cardiac patches. In this approach, a biopsy of fatty tissue was taken from patients and the cellular and a‐cellular materials were separated. While the cells were reprogrammed to become pluripotent stem cells, the extra‐cellular matrix (ECM) was processed into a personalized hydrogel. Following mixture of the cells and the hydrogel, the cells were efficiently differentiated to cardiac cells to create patient‐specific, immunocompatible cardiac patches.^9^ However, these cardiac patches did not contain blood vessel networks that match the anatomical architecture of the patient's vasculature. This pre‐engineered vasculature within the parenchymal tissue was previously shown to be critical for patch survival and function after transplantation.^10^, ^11^, ^12^, ^13^, ^14^, ^15^, ^16^, ^17^

In recent years, the strategy of 3D tissue printing evolved,^18^, ^19^, ^20^ allowing to create vasculature within hydrogels.^21^ However, in most of the studies, the endothelial cells (ECs) that form the blood vessels were printed without the parenchymal tissue, which was later on casted on top of the vessels.^22^ In other pioneering works, the researchers were able to print ECs together with thin surrounding tissues.^22^ However, the obtained tissues were not thick, the ECs did not form open blood vessels and perfusion through them was not demonstrated. Different strategies include printing of the parenchymal tissue with open, a‐cellular channels in‐between, followed by external perfusion of ECs to form the blood vessels.^23^, ^24^, ^25^ Finally, decellularized hydrogels were also used for printing nonvascularized tissues.^19^, ^26^ Therefore, to the best of our knowledge, the aforementioned studies did not demonstrate printing of a full, thick vascularized patch in one step.

**Figure 1:**
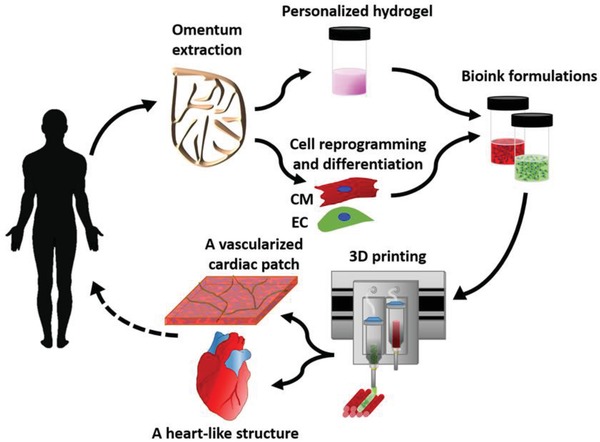
Concept schematic. An omentum tissue is extracted from the patient and while the cells are separated from the matrix, the latter is processed into a personalized thermoresponsive hydrogel. The cells are reprogrammed to become pluripotent and are then differentiated to cardiomyocytes and endothelial cells, followed by encapsulation within the hydrogel to generate the bioinks used for printing. The bioinks are then printed to engineer vascularized patches and complex cellularized structures. The resulting autologous engineered tissue can be transplanted back into the patient, to repair or replace injured/diseased organs with low risk of rejection.

Here, we report on the development and application of advanced 3D printing techniques using the personalized hydrogel as a bioink. In this strategy, when combined with the patient own cells, the hydrogel may be used to print thick, vascularized, and perfusable cardiac patches that fully match the immunological, biochemical and anatomical properties of the patient. Furthermore, we demonstrate that the personalized hydrogel can be used to print volumetric, freestanding, cellular structures, including whole hearts with their major blood vessels (**Figure** **1**).

## 2 Results and Discussion

**Figure 2:**
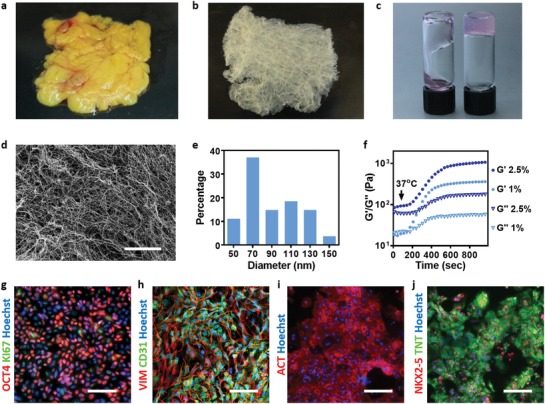
Bioinks characterization. A human omentum a) before and b) after decellularization. c) A personalized hydrogel at room temperature (left) and after gelation at 37 °C (right). d) A SEM image of the personalized hydrogel ultrastructural morphology, and e) a histogram of the fibers diameter. f) Rheology measurements of 1% w/v and 2.5% w/v omentum hydrogels, showing the gelation process upon incubation at 37 °C. g) Stromal cells originated from human omental tissues were reprogrammed to become pluripotent stem cells (red: OCT4, green: Ki67 and blue: nuclei). h) Differentiation to ECs as determined by CD31 (green) and vimentin staining (red). Differentiation to cardiac lineage: i) staining for sarcomeric actinin (red), j) staining for NKX2‐5 (red), and TNNT2 (green). Scale bars: (e) = 10 µm, (g,i,j) = 50 µm, (h) = 25 µm.

Omental tissues from humans (**Figure** **2**a) or pigs were obtained. While cells were extracted from one piece of the tissue, the remaining material was decellularized (Figure 2b), as previously described,^9^ and processed to generate a 2.5% (w/v) thermoresponsive hydrogel, serving as a bioink for 3D printing (Figure 2c). The bioink, composed of collagenous nanofibers (Figure 2d,e) behaved as a weak gel at room temperature (RT) and became stronger upon heating to 37 °C (Figure 2f).^9^, ^27^

Our experiments included two cellular models. One model was used as a proof‐of‐concept for the patient‐specific treatment, and included induced pluripotent stem cells (iPSCs)‐derived cardoimyocytes (CMs) and ECs. The second model relied on rat neonatal CMs, human umbilical vein endothelial cells (HUVECs) and lumen‐supporting fibroblasts, allowing large‐scale printing. Two cellular bioinks were generated. The cardiac cell bioink, which was used for printing the parenchymal tissue, and a bioink consisting of blood vessel‐forming cells.

To this end, iPSCs were reprogrammed from the stromal cells of human omental tissues, used for hydrogels preparation. The cells exhibited the pluripotency markers OCT4 and Ki67 (Figure 2g), and after exposure to endothelial and cardiac cells differentiation protocols, as previously described,^9^ expressed CD31 and vimentin (Figure 2h) and NKX2‐5, sarcomeric actinin, and troponin (Figure 2i,j).

**Figure 3:**
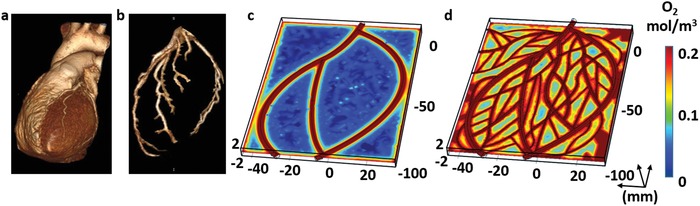
Imaging of the heart and patch modeling. CT image of a) a human heart and b) left ventricle coronary arteries. c) A model of oxygen concentration profile in an engineered patch. d) Replanning of the model showed better oxygen diffusion, sufficient to support cell viability.

We next sought to fit the 3D printing scheme to the anatomy of a patient. Computerized tomography (CT) of a patient's heart (**Figure** **3**a) was used to identify the 3D structure and orientation of the major blood vessels in the left ventricle (Figure 3b). In order to fit a 3D cardiac patch to the patient's left ventricle, the patch dimensions and the blood vessel geometry were designed by computer‐aided design (CAD) software that used anatomical data from the CT images. CT cannot provide images of small blood vessels, therefore, to ensure adequate exposure of the entire cardiac patch to oxygenated medium during in vitro cultivation and after transplantation, smaller blood vessels were added to the basic vasculature design, according to a mathematical model. The model took into consideration oxygen diffusion according to Fick's second law and consumption according to Michaelis–Menten equation,^28^ allowing to design an optimal size, distribution, and orientation of the supplemented blood vessels (Figure 3c,d; Figure S1, Supporting Information).

**Figure 4:**
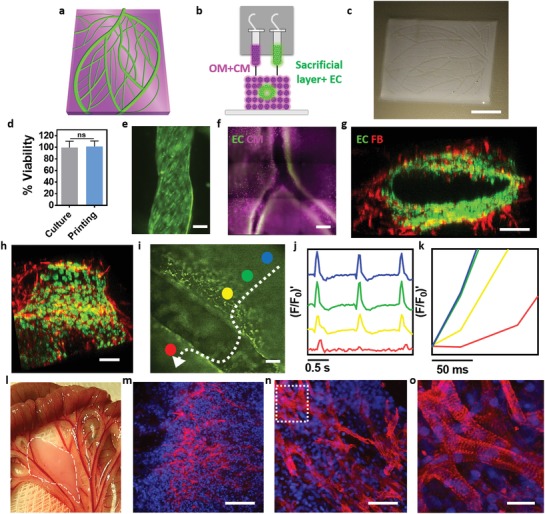
3D printing of personalized cardiac patches. a) A 3D model of the cardiac patch. b) A side view of the printing concept and the distinct cellular bioinks. c) A printed vascularized cardiac patch. d) Cell viability after printing. e) A printed blood vessel, continuously layered with GFP‐expressing ECs. f) A printed iPSCs‐derived cardiac patch where the blood vessels (CD31 in green) are seen in‐between the cardiac tissue (actinin in pink). g,h) Cross‐sections of a single lumen, showing the interactions of GFP‐expressing ECs and RFP‐expressing fibroblasts. i–k) Calcium imaging within a printed vascularized cardiac patch (separate regions of interest are represented in different colors. The white arrow represents signal direction). i) The lumen of a blood vessel can be easily observed in‐between the cardiac cells. j,k) Quantification of calcium transients across a lumen of the vascularized cardiac patch. l) Transplantation of the printed patch in between two layers of rat omentum. Dashed, white line highlights the borders of the patch. m–o) Sarcomeric actinin (red) and nuclei (blue) staining of sections from the explanted patch (panel (o) represents a high magnification of the marked area in (n). Scale bars: (c) = 1 cm, (e,g,h,l,m) = 100 µm, (f) = 500 µm, (n) = 50 µm, (o) = 25 µm.

The personalized hydrogel was next mixed either with iPSC‐derived CMs or neonatal cardiac cells, and was used to print the engineered cardiac tissue. iPSC‐derived ECs or a combination of mature GFP‐expressing human ECs with RFP‐expressing fibroblasts were mixed with gelatin, which served as a sacrificial bioink. A 3D printer, equipped with extrusion‐based print heads, was used to print the parenchymal cell‐containing hydrogel simultaneously with gelatin that contains blood vessel‐forming cells (**Figure** **4**a,b) to create a thick (≈2 mm), 3D, patient‐specific, vascularized patch that had high cell viability (Figure 4c,d; Movie S1, Supporting Information). Upon incubation at 37 °C, the blood vessel‐forming cells adhered to the edges of the omentum hydrogel, while the gelatin became liquid and was washed away from the construct, leaving open cellular lumens of ≈300 µm in diameter within the cardiac patches (Figure 4e–h; Figure S2, Supporting Information). The cardiac patches were physically robust and could be handled easily so that they could be pulled out and returned to the medium without loss of shape (Movie S2 and Figure S3a, Supporting Information). Furthermore, liquid could be infused into the open lumens of the patches, indicating that their lumen structure was maintained (Movie S3 and Figure S3b, Supporting Information). On day 7, the ECs formed a continuous layer at the edges of the hydrogel (Figure 4e,h), and the iPSC‐derived patches were stained against actinin and CD31 in order to detect CMs and ECs, respectively (Figure 4f). Furthermore, the interactions of GFP‐expressing ECs and RFP‐expressing fibroblasts (for lumen stabilization) within the forming lumen were evaluated by a confocal microscopy (Figure 4g,h; Movie S4 and Figure S4, Supporting Information).

Next, the function of the printed vascularized cardiac patch was evaluated by measuring calcium transients in the contracting engineered tissue. Signal propagation exceeded 10 cm s^−1^ at the parenchymal tissue, however a short delay in propagation could be detected across a 300 µm blood vessel, probably due to the detour of the signal around the vessel (Figure 4i–k; Movies S5–S7, Supporting Information). Finally, to assess the presence and morphology of the printed cells in vivo, the engineered patches were transplanted and secured between two layers of rat omentum (Figure 4l). Seven days later, the printed patches were located using a fluorescence prestaining of the CMs. The cellularized patches were then extracted, fixed, sectioned, and stained against sarcomeric actinin. As shown, the cells were elongated and aligned, with massive striation, which indicated on their contractility potential (Figure 4m–o; Movie S8, Supporting Information). Furthermore, the printed lumen could be seen within the tissue by locating the fluorescent ECs (Figure S5, Supporting Information).

Overall, taken together, these results demonstrate the ability to use the patient's own cells and materials in 3D printing of fully personalized, contracting cardiac patches that closely fit to the patient's biochemical and cellular properties, as well as for the anatomy of the patient. This approach can yield several mm thick structures, which is sufficient for engineering clinically relevant cardiac patches.

However, when printing whole organs or tissues with significantly larger dimensions and high complexity is required, the patient‐specific hydrogel cannot sustain its weight, and a different printing strategy is needed.

**Figure 5:**
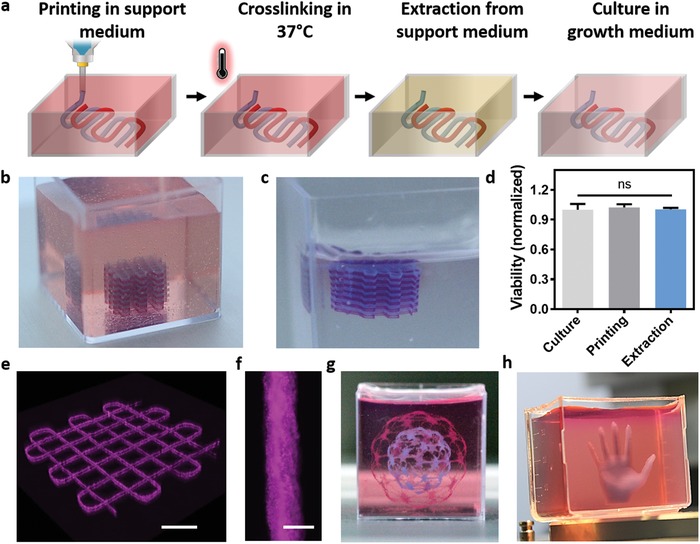
Printing the personalized hydrogel in a supporting medium. a) A scheme of the 3D printing concept. The construct is free‐formed printed inside the support followed by incubation at 37 °C to crosslink the personalized hydrogel. Then, the structure can be safely extracted by an enzymatic or chemical degradation process of the support material and transferred into growth medium for culturing. b) A multilayered crisscross construct printed inside the support bath, and c) after its extraction. d) Cell viability before and after printing and after extraction. e) 3D confocal image of a double layered construct, printed in the support medium. f) A single strand of the personalized hydrogel within the support. g,h) Accurate, high resolution thick structures printed from the personalized hydrogel. Scale bars: (b) = 0.5 cm, (e) = 1 mm, (f) = 100 µm, (g,h) = 1 cm.

Therefore, we next relied on the concept of printing in a supporting medium.^20^ Previously, Hinton et al. developed a semi‐transparent support medium composed of gelatin microparticles, which allowed to print embryonic heart structure that did not contained cells.^29^ Such strategy could support free‐form printing of structures composed of a variety of bioinks. However, as the extraction procedure of the support medium was by quick melting of the gelatin microparticles at 37 °C, such strategy would not allow long‐term support until the personalized hydrogel is fully cured. Other strategies include the use of synthetic, transparent granular support for printing cellular structures.^30^ However, in this case the integrity of delicate structures and viability of sensitive cells can be jeopardized by the mechanical force needed for removal of the support medium. Inspired by these two elegant, pioneering works, we sought to formulate a support medium that better fits the printing of the personalized hydrogel. To this end, we developed a fully transparent, cell‐friendly, microparticulate formulation that allows free‐form printing and curing in a wide range of temperatures, and extraction by a controllable and delicate process (**Figure** **5**a). This support material is composed of alginate microparticles in xanthan gum‐supplemented growth medium (Figure S6, Supporting Information) that can undergo safe enzymatic or chemical degradation for extraction (Figure 5b,c), while maintaining high cell viability (Figure 5d). Using this printing approach we were able to print accurate, high resolution thick structures from the personalized hydrogel (Figure 5e–h; Figure S7 and Movies S9–S11, Supporting Information).

**Figure 6:**
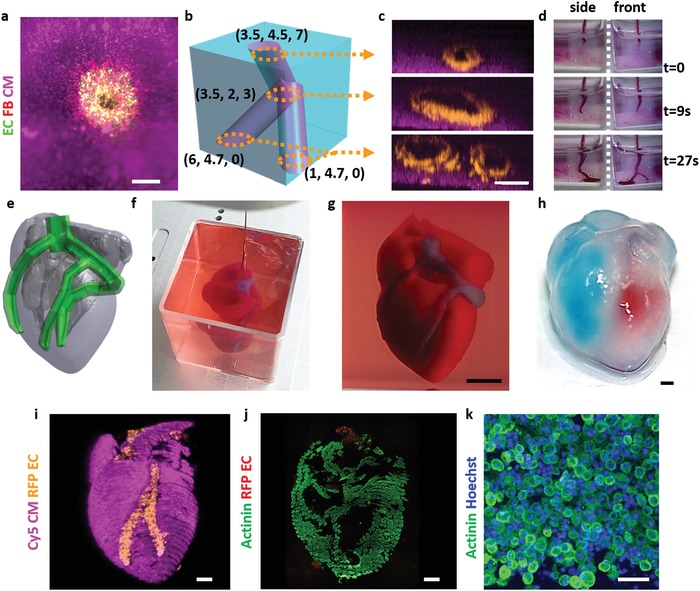
Printing thick vascularized tissues. a) A top view of a lumen entrance (CD31; green) in a thick cardiac tissue (actinin; pink). b) A model of a tripod blood vessel within a thick engineered cardiac tissue (coordinates in mm), and c) the corresponding lumens in each indicated section of the printed structure. d) Tissue perfusion visualized from dual viewpoints. e–k) A printed small‐scaled, cellularized, human heart. e) The human heart CAD model. f,g) A printed heart within a support bath. h) After extraction, the left and right ventricles were injected with red and blue dyes, respectively, in order to demonstrate hollow chambers and the septum in‐between them. i) 3D confocal image of the printed heart (CMs in pink, ECs in orange). j,k) Cross‐sections of the heart immunostained against sarcomeric actinin (green). Scale bars: (a,c,h, i,j) = 1 mm, (g) = 0.5 cm, (k) = 50 µm.

Following, we have utilized the technology to print blood vessels within thick tissues. Here, both cardiac and endothelial cells were mixed with the personalized hydrogel to create two distinct bioinks. Each layer of the printed construct consisted of the parenchymal tissue and the blood vessel‐forming cells, resulting in a thick vascularized tissue (Figure S8, Supporting Information). In this manner, perfusable patches with an actual triaxial lumen were printed. A top view of the entry to one lumen (green) within the cardiac tissue (pink) could be clearly observed by a confocal microscope (**Figure** **6**a; Movie S12, Supporting Information). As the immunostaining of thick cellular constructs (7 × 7 × 7 mm) is not efficient for visualizing the core of the tissue, we next supplemented the bioinks with fluorescently dyed nanoliposomes (pink for the parenchymal tissue and orange for the blood vessels). As shown, the triaxial lumen within the thick structure could be visualized (Figure 6b,c) and dye could be infused, indicating on the potential of the printed blood vessels to efficiently transfer blood (Figure 6d; Movie S13, Supporting Information).

Next, we sought to demonstrate the ability of the approach to print volumetric, complex anatomical architectures. As a proof of concept, we have fabricated small‐scale cellularized human hearts with major blood vessels, based on a digital design (Figure 6e). The hearts (height: 20 mm; diameter: 14 mm) were printed within the support medium with two distinct bioinks, containing Cy5‐prestained CMs or RFP‐expressing ECs. For better visualization, constructs could be printed with bioinks supplemented with 1 µm blue (blood vessels) and red (cardiac tissue) polystyrene spheres (Figure 6f,g; Figure S9 and Movie S14, Supporting Information). Following heating to 37 °C and extraction, blue and red dyes were injected into the right and left ventricles, respectively, to demonstrate the integrity of the different compartments (Figure 6h). The ability to manipulate and perfuse the printed hearts was also demonstrated, indicating on their basic anatomical structure and mechanical stability and robustness (Movies S15 and S16, Supporting Information). The mechanical properties of the printed heart‐structured ECM revealed a close resemblance to the properties of decellularized rat hearts (Figure S10, Supporting Information), indicating on the suitability of the printed hydrogel to serve as a scaffolding material for this purpose. 3D confocal image of the printed heart reveals the initial spatial organization of the CMs and ECs, according to the layers of the printing plan (Figure 6i; Movies S17 and S18 and Figure S11, Supporting Information). One‐day post printing, a cross‐section of the printed heart was stained against sarcomeric actinin (green) to reveal the internal compartmental structure (Figure 6j). Higher magnification of the cells comprising the printed heart showed homogeneous distribution of the CMs (Figure 6k). Future studies should focus on development of designated hardware and procedures to enable efficient and controlled cultivation of the printed organ. Long‐term culturing that provides the tissue with a constant supply of oxygen and nutrients, as well as biochemical, physiological, and electromechanical cues will allow natural maturation processes to take place. Among these processes are the proper assembly of muscle bundles with elongated cells and massive striation, as well as ECM remodeling. Both are essential for generation of functional, mechanically stable organ that may be amenable to clinical applications.

## 3 Conclusion

Overall, in this paper we demonstrate, for the first time, the use of fully personalized, nonsupplemented materials as bioinks for 3D printing. In this approach, a fatty tissue is extracted from the patient and the cellular and a‐cellular materials are processed to form diverse personalized bioinks. We report on the potential of the technology to engineer vascularized cardiac patches that fully match the anatomical structure, as well as the biochemical and cellular components of any individual. As we have previously shown, since the bioinks originated from the same patient, the engineered patches will not provoke an immune response after transplantation, eliminating the need for immunosuppression treatment.^9^ Furthermore, a customized formulation was developed to support the free‐form printing of the personalized hydrogel, allowing to fabricate large, complex biological structures. Thus, cellularized hearts with a natural architecture were engineered, demonstrating the potential of the approach for organ replacement after failure, or for drug screening in an appropriate anatomical structure. Long‐term in vitro studies and in vivo implantation experiments in animal models should be conducted in order to adequately evaluate the fate and therapeutic value of the printed tissues.

Although 3D printing is considered a promising approach for engineering whole organs, several challenges still remain.^18^ These include efficient expansion of iPSCs to obtain the high cell number required for engineering a large, functioning organ. Additionally, new bioengineering approaches are needed to provide long‐term cultivation of the organs and efficient mass transfer, while supplying biochemical and physical cues for maturation. The printed blood vessel network demonstrated in this study is still limited. To address this challenge, strategies to image the entire blood vessels of the heart and to incorporate them in the blueprint of the organ are required. Finally, advanced technologies to precisely print these small‐diameter blood vessels within thick structures should be developed.

## 4 Experimental Section

*iPSCs Culture*: iPSCs were generated from omental stromal cells and were a kind gift from Dr. Rivka Ofir, Ben Gurion University. The undifferentiated cells were cultivated on culture plates, pre coated with Matrigel (BD, Franklin Lakes, New Jersey), diluted to 250 µg mL^−1^ in Dulbecco's modified Eagle medium (DMEM)/F12 (Biological Industries, Beit HaEmek, Israel). Cells were maintained in NutriStem (Biological Industries) medium containing 1% penicillin/streptomycin (Biological Industries) and cultured under a humidified atmosphere at 37 °C with 5% CO_2_. Medium was refreshed daily and cells were passaged weekly by treatment with 1 U mL^−1^ dispase (Stemcell Technologies, Vancouver, Canada) followed by mechanical trituration.

*Cardiomyocyte Differentiation from iPSCs*: Cells were differentiated as previously described.^9^, ^31^ Briefly, growth media (NutriStem) was refreshed daily until iPSCs reached 100% confluence. At this point (day 0) medium was changed to RPMI (Biological Industries) supplemented with 0.5% glutamine (Biological Industries), B27 minus Insulin (X50, Invitrogen, Carlsbad, California) and 10 × 10^−6^ m CHIR‐99021 (Tocris, Bristol, UK). Medium was refreshed every other day. At day 2, CHIR‐99021 was removed from media. At day 4, 5 × 10^−6^ m IWP‐2 (Tocris) was added to media and was removed on day 6. At day 8, contracting implants were observed and medium was changed to medium supplemented with 0.5% glutamine, B27 minus retinoic acid (×50, Invitrogen), and 1 × 10^−6^ m retinoic acid (Sigma‐Aldrich). After day 10, medium was changed to M‐199 (Biological Industries), supplemented with 0.1% penicillin/streptomycin, 5% fetal bovine serum (FBS, Biological Industries), 0.6 × 10^−3^ m CuSO4 · 5H2O, 0.5 × 10^−3^ m ZnSO4 · 7H2O, 1.5 × 10^−6^ m vitamin B12 (Sigma‐Aldrich), this media was refreshed every other day.

*Endothelial Cell Differentiation from iPSCs*: Cells were differentiated as previously described with modifications.^9^, ^32^ Briefly, After iPSCs reached ≈90% confluence (day 0), medium was changed to 50% (v/v) Neurobasal (Invitrogen) 50% (v/v) DMEM/F12 (Biological Industries), supplemented with 1% penicillin/streptomycin, 1% glutamine, B27 minus retinoic acid, N2 supplement (×100, Invitrogen), 1% nonessential amino acids (Invitrogen), 10 × 10^−6^ m β‐mercaptoethanol (Gibco, Welltham, Massachusetts), 8 × 10^−6^ m CHIR‐99021 (Tocris), and BMP4 20 ng mL^−1^ (R&D, Minneapolis, Minnesota). On day 3, medium was changed to EGM‐2 (Lonza, Basel, Switzerland), supplemented as according to the manufacturer instructions, and was refreshed every other day.

*Cell Dissociation from Matrigel‐Coated Plates*: Cells grown on Matrigel‐coated plates were dissociated by enzyme digestion with collagenase type II (95 U mL^−1^, Worthington, Lakewood, New Jersey) and pancreatin (0.6 mg mL^−1^, Sigma‐Aldrich) in DMEM (37 °C, 30 min), followed by TrypLE express (STEMCELL) treatment.

*Mathematical Modeling*: Anonymous CT Images of a human heart have been contributed by the courtesy of Tel Aviv Sourasky Medical Center, Israel. The digital data file was then analyzed using RadiAnt DICOM viewer (Medixant). The left ventricle major blood vessels were segmented and measured. Based on these measurements, a 3D model of the cardiac patch was generated using COMSOL Multiphysics software. Oxygen concentration profile was calculated based on Fick's second law, Michaelis–Menten equations and the following data: Maximum cellular O^2^ consumption rate of 5.44 × 10^−2^ nmol s^−1^ cm^−3^, Michaelis–Menten constant for oxygen consumption of 3.79 nmol cm^−3^, diffusion coefficient (oxygen in hydrogel) of 1 × 10^−9^ m^2^ s^−1^ (Figure S1c,d). The model was then supplemented with blood vessels, ensuring that no region reach critical oxygen concentration (2.64 × 10^−3^ mol m^−3^).^28^, ^33^

*Fluorescent Endothelial and Fibroblast Cell Culture*: Red fluorescent protein‐expressing human neonatal dermal fibroblast (RFP‐HNDF) cells (Angio‐Proteomie, Boston, Massachusetts) were grown in DMEM supplemented with 10% FBS, 1% penicillin/streptomycin, 1% glutamine, 1% nonessential amino acids, and 0.2% β‐mercaptoethanol. Green/red fluorescent protein‐expressing primary human umbilical vein endothelial cells (GFP/RFP‐HUVECs, Angio‐Proteomie) were maintained in EGM‐2.

*Neonatal Cardiac Cell Isolation*: Neonatal cardiac cells were isolated according to Tel Aviv University ethical use protocols from intact ventricles of 1‐ to 3‐day‐old neonatal Sprague‐Dawley rats as previously reported.^34^ Cells were isolated using 6 cycles (37 °C, 30 min each) of enzyme digestion with collagenase type II (95 U mL^−1^) and pancreatin (0.6 mg mL^−1^) in DMEM. After each round of digestion, cells were centrifuged (600 g, 5 min) and resuspended in M‐199 culture medium supplemented with 0.6 × 10^−3^ m CuSO4 · 5H2O, 0.5 × 10^−3^ m ZnSO4 · 7H2O, 1.5 × 10^−3^ m vitamin B12, 500 U mL^−1^ penicillin, and 100 mg mL^−1^ streptomycin, and 0.5% FBS. To enrich the cardiomyocyte population, cells were suspended in culture medium with 5% FBS and were pre‐plated twice for 45 min. Cell number and viability were determined by a hemocytometer and trypan blue exclusion assay.

*Bioinks Preparation*: Omenta were decellularized as previously described.^9^ Briefly, human omenta (Helsinky #0237‐16‐ASF, Assaf Harofeh Medical Center, Israel; a consent was obtained from all subjects), or omenta from the remains of healthy pigs (Kibutz Lahav – designated for the food industry), were washed with phosphate buffered saline (PBS) (at least three human and ten pig omenta were used). Then, transferred to hypotonic buffer (10 × 10^−3^ m Tris, 5 × 10^−3^ m ethylenediaminete‐traacetic acid (EDTA), and 1 × 10^−6^ m phenylmethanesulfonyl‐fluoride, pH 8.0) for 1 h. Next, tissues were frozen and thawed three times in the hypotonic buffer. The tissues were washed gradually with 70% (v/v) ethanol and 100% ethanol for 30 min each. Lipids were extracted by three, 30 min washes of 100% acetone, followed by 24 h incubation in a 60/40 (v/v) hexane: acetone solution (solution was exchanged three times in 24 h). The defatted tissue was washed in 100% ethanol for 30 min and incubated over‐night (O.N.) at 4 °C in 70% ethanol. Then, the tissue was washed four times with PBS (pH 7.4) and incubated in 0.25% Trypsin‐EDTA solution (Biological Industries) O.N. The tissue was washed thoroughly with PBS and incubated in 1.5 m NaCl (solution was exchanged three times in 24 h), followed by washing in 50 × 10^−3^ m Tris (pH 8.0), 1% triton‐X100 (Sigma‐Aldrich) solution for 1 h. The decellularized tissue was washed in PBS followed by double distilled water and then frozen (–20 °C) and lyophilized. The dry, decellularized omentum was ground into powder (Wiley Mini‐Mill, Thomas Scientific, Swedesboro, NJ). The milled omentum was then enzymatically digested for 96 h at RT with stirring, in a 1 mg mL^−1^ solution of pepsin (Sigma‐Aldrich, 4000 U mg‐1) in 0.1 M HCl. Subsequently, pH was adjusted to 7.4 using 5 m NaOH and either DMEM/F12 × 10 or PBS ×10 (Biological industries). The final concentration of decellularized omentum in the titrated solution was 1% (w/v). For the personalized bioink preparation, omentum gel 1% (w/v) was homogenized at 15 000 rpm for 2 min (Silent Crusher‐M with 8F generator probe, Heidolph Brinkmann, Schwabach, Germany) and then weighted. Subsequently, while constantly stirred, the gel was allowed to reduce under a jet of sterile air until reached 1/3 of its initial weight. The concentrated gel (2.5% w/v) was then centrifuged at 300 g for 2 min to remove air bubbles and stored at 4 °C until use. Dissociated iPSC derived CMs or neonatal rat cardiac cells were then dispersed in M‐199 medium and mixed with the omentum gel, reaching a final hydrogel concentration of 1% w/v with cells concentration of 1 × 10^8^ mL^−1^. The cell‐laden ink was loaded into a syringe and kept at 4 °C. Sacrificial ink: Gelatin hydrogel was prepared by dissolving 15% w/v gelatin (from porcine skin, type A, Sigma‐Aldrich) in 40 °C warmed EBM‐2 (Lonza). The solution was then filtered by 0.22 µm syringe filter and kept at 4 °C until further use. Cell‐laden gelatin ink was generated by dispersing ECs in warm EGM‐2 medium, mixed with prewarmed gelatin ink at a 1:2 v/v ratio, reaching a final concentration of 10% w/v gelatin and 1.5 × 10^7^ cells mL^−1^. HNDF cells were added to the bioink to a final concentration of 3 × 10^6^ cells mL^−1^. The cell‐laden ink was then loaded into a syringe and allowed to cool to room temperature (22 °C).

In the support bath method, in order to form the blood vessel perimeters, the personalized hydrogel bioink was used, encapsulating ECs at a concentration of 2 × 10^7^ cell mL^−1^.

*Support Medium Preparation*: For the generation of the printing support medium, an aqueous solution containing 0.32% (w/v) sodium alginate (PROTANAL LF 200 FTS, a generous gift from FMC BioPolymer), 0.25% (w/v) Xanthan gum (XANTURAL 180, kindly provided by CP Kelco), and 9.56 × 10^−3^ m calcium carbonate (as suspension, Sigma‐Aldrich) was prepared. While constantly stirred, the mixture was supplemented with freshly prepared, predissolved d‐(+)‐gluconic acid δ‐lacton (Sigma‐Aldrich) to reach a final concentration of 19.15 × 10^−3^ m. This results in a slow decrease in the pH and solubilization of the calcium carbonate and liberation of the calcium ion that crosslinks the alginate. When the solution's viscosity is increased to a level that prevents precipitation of the calcium carbonate, the stirring was stopped and the mixture was incubated at RT for 24 h. Double distilled water at four times the volume of the resulted hydrogel were then added, followed by homogenization at 25 000 rpm (HOG‐020 homogenizer with GEN‐2000 generator probe, MRC ltd, Israel). The homogenate was centrifuged at 15 800 *g* for 20 min. The pellet was resuspended in DMEM/F12 (HAM) 1:1 culture media (Biological Industries) and centrifuged again, after which the supernatant was discarded. The pellet was then supplemented with 1% (w/v) xanthan gum in DMEM/F12 (HAM) 1:1 media (reaching a final concentration of 0.1%) followed by vigorous vortexing to homogenize the mixture.

*Cardiac Patches Printing Process*: Cardiac patches were printed using 3DDiscovery printer (regenHU, Villaz‐Saint‐Pierre, Switzerland). The bioinks were extruded through 30G needles onto glass slides. First, the CMs cell laden omentum gel was extruded in a crisscross geometry, creating the two lower layers of the patch. The third layer was composed of omentum gel, creating the supporting walls between which ECs laden gelatin ink was deposited to generate the vascular network. On top, two layers of crisscross CMs cell laden omentum gel were extruded, encapsulating the printed vessels. The printed patches were then incubated at 37 °C for 30 min to crosslink the omentum gel and to liquefy the gelatin, followed by submerging in EGM‐2 media for further culturing.

*Printing in a Support Bath*: Support medium was transferred into a transparent, open sterile plastic box immediately prior to printing. The a‐cellularized or cellularized constructs were then printed (3D Discovery printer) by extrusion (through 30G needles) according to designs generated by BioCAD drawing software (regenHU) or according to data from STL files (sliced and processed by BioCAM software (regenHU)), which were downloaded from Thingiverse (www.thingiverse.com) (“Spheres in sphere” by Syzguru11 (modified), under the Creative Commons – Attribution license‐ CC BY 3.0 – https://creativecommons.org/licenses/by/3.0/; “Hand” by Teak (unmodified), under the Creative Commons – Attribution license – CC BY 3.0 https://creativecommons.org/licenses/by/3.0/; “Anatomical Human Heart” by 517860 (modified), under the Creative Commons – Attribution – Share Alike license – CC BY‐SA 3.0 https://creativecommons.org/licenses/by‐sa/3.0/). The cellularized constructs were printed using two omentum bioinks containing CMs and ECs. To improve visualization, constructs could be printed with bioinks supplemented with 1 µm blue or red polystyrene microparticles (Sigma‐Aldrich) or with lipid nanoparticles encapsulating cy3 or cy5 molecules, which were a kind gift from Prof. Dan Peer, Tel Aviv University. Upon completion of the printing process, the box was incubated at 37 °C for 45 min to crosslink the personalized hydrogel. Then, support medium was gradually aspirated and replaced with EGM‐2 medium containing alginate lyase 1 U mL^−1^ (Sigma‐Aldrich). The printed construct was then cultured O.N., allowing final, complete degradation of the alginate particles. Finally, the medium was changed to fresh EGM‐2 medium for further culturing.

*Rheological Properties*: Rheological measurements (*n* = 3) were taken using Discovery HR‐3 hybrid Rheometer (TA Instruments, DE) with 8 mm diameter parallel plate geometry with a Peltier plate to maintain the sample temperature. The samples were loaded at a temperature of 4 °C, which was then raised to 37 °C to induce gelation; during which the oscillatory moduli of samples were monitored at a fixed frequency of 0.8 rad s^−1^ and a strain of 1%. Compression tests on the printed or decellularized^35^ hearts were performed with 20 mm diameter parallel plate geometry which compressed the samples at a rate of 5 µm s^−1^.

*Immunostaining, Confocal Imaging, and Calcium Imaging*: Cells/tissues were fixed in 4% formaldehyde, permeabilized with 0.05% (v/v) triton X‐100 and blocked with PBS, 1% bovine serum albumin, 10% FBS, and stained with primary antibodies followed by secondary antibodies (as indicated in the antibody list). Cells/tissues were imaged using an upright confocal microscope (Nikon ECLIPSE NI‐E) and inverted fluorescence microscope (Nikon ECLIPSE TI‐E). Images were processed and analyzed using NIS elements software (Nikon Instruments). Representative images from at least three different experiments were chosen. For calcium imaging, the cardiac patches were incubated with 10 × 10^−6^ m fluo‐4 AM (Invitrogen) and 0.1% Pluronic F‐127 (Sigma‐Aldrich) for 45 min at 37 °C. Cardiac patches were then washed in culture medium and imaged using an inverted fluorescence microscope. Videos were acquired with an ORCA‐Flash 4.0 digital complementary metal‐oxide semiconductor camera (Hamamatsu Photonics) at 100 frames s^−1^.

*Antibody and Dyes List*: Antibodies for NKX2‐5 (ab91196, 1:500), Troponin (ab47003, 1:100), CD31 (ab32457, 1:100), OCT4 (ab27985, 1:100), Ki67 (ab16667, 1:250), and Cytopainter deep red (ab138894) were acquired from Abcam (Cambridge, MA). Antibodies for actinin (A7811, 1:500) were acquired from Sigma‐Aldrich. Antibodies for Vimentin (1117481A, 1:100) were acquired from Invitrogen. Secondary antibodies: FITC‐conjugated goat anti‐mouse (ab6785, 1:800) and Alexa Flour 555‐conjugated goat anti‐mouse (ab150118, 1:500) have been acquired from Abcam. Alexa 647‐conjugated goat anti‐mouse (115‐605‐003, 1:500) and Alexa Fluor 488‐conjugated goat anti‐rabbit (111‐545‐144, 1:500) have been acquired from Jackson (Pennsylvania). For nuclei detection, the cells were incubated for 3 min with Hoechst 33258 (1:100) (Sigma‐Aldrich).

*Viability Assay*: Cell viability was determined using a Live/Dead fluorescent staining with fluorescein diacetate (Sigma‐Aldrich, 7 µg mL^−1^) and propidium Iodide (Sigma‐Aldrich, 5 µg mL^−1^) for 10 min at 37 °C. The number of live and dead cells was determined by manual counting using NIS Elements software (Nikon) from at least three different microscopic field (*n* ≥ 3 in each experiment), visualized by inverted fluorescence microscope.

*Scanning Electron Microscopy (SEM)*: Human omentum hydrogel samples were fixed with 2.5% glutaraldehyde (24 h at 4 °C), followed by graded incubation series in ethanol–water solutions (25–100% (v/v)). All samples (*n* ≥ 3) were critical point dried, sputter‐coated with gold in a Polaron E 5100 coating apparatus (Quorum technologies, Lewis, UK) and observed under JSM‐840A SEM (JEOL, Tokyo, Japan).

*Statistical Analysis*: Statistical analysis data are presented as means ± s.d. Differences between samples were assessed by student's *t*‐test. *p* < 0.05 was considered significant. ns denotes not significant. Analyses were performed using GraphPad prism version 6.00 for windows (GraphPad Software).

## Conflict of Interest

The authors declare no conflict of interest.

## Supporting information

Supplementary

Supplementary

Supplementary

Supplementary

Supplementary

Supplementary

Supplementary

Supplementary

Supplementary

Supplementary

Supplementary

Supplementary

Supplementary

Supplementary

Supplementary

Supplementary

Supplementary

Supplementary

Supplementary
