# Supplementary material for: 3D Printing of Personalized Thick and Perfusable Cardiac Patches and Hearts
Source: Adv Sci (Weinh). 2019 Apr 15;6(11):1900344. doi: 10.1002/advs.201900344 (PMC6548966; doi:10.1002/advs.201900344)
Supplement: Supplementary file 1 — Supplementary [file ADVS-6-1900344-s002.pdf]

Copyright WILEY-VCH Verlag GmbH & Co. KGaA, 69469 Weinheim, Germany, 2019.

Supporting Information

for *Adv. Sci.,* DOI: 10.1002/advs.201900344

3D Printing of Personalized Thick and Perfusable Cardiac Patches and Hearts

*Nadav Noor, Assaf Shapira, Reuven Edri, Idan Gal, Lior Wertheim, and Tal Dvir**

Supplementary Information for

3D printing of personalized thick and perfusable cardiac patches and hearts

Nadav Noor[1,2,†] Assaf Shapira[1,†] Reuven Edri[1,†], Idan Gal[1], Lior Wertheim[1,2] and Tal Dvir[1,2,3,4,*]

[1]The School for Molecular Cell Biology and Biotechnology, Faculty of Life Sciences, Tel Aviv University, Tel Aviv 6997801, Israel.

[2]Department of Materials Science and Engineering, Faculty of Engineering, Tel Aviv University, Tel Aviv 6997801, Israel.

[3]The Center for Nanoscience and Nanotechnology, Tel Aviv University, Tel Aviv 6997801, Israel

[4]Sagol Center for Regenerative Biotechnology, Tel Aviv University, Tel Aviv 6997801, Israel

†Equal contribution

*Correspondence to Tal Dvir (tdvir@tauex.tau.ac.il)

This PDF file includes:

Materials and Methods
Supplement Figures. **1-11**
Captions for Movies **1** to **18**

Other Supplementary Information for this manuscript includes the following:

Supplementary Movies **1** to **18**

Supplementary Fig. 1. Modeling of the cardiac patch before and after addition of blood vessels to optimize mass transfer within the printed structure.

a b

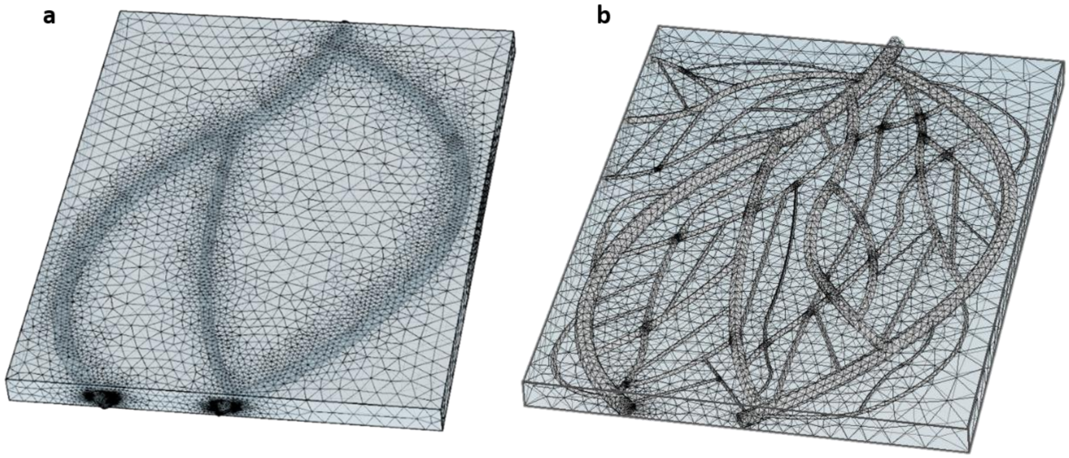

c

$$r = \frac{Vmax \times S}{K_m + S}$$

d

$$\frac{dC_a}{dt} + \nabla(-D_o \nabla C_a) = r$$

(a) The mesh used for the mathematical modeling of the patch, based on the anatomical data from the CT images. (b) The mesh of the blood vessels-supplemented model. (c) Michalis-Menten equation used to calculate oxygen cellular consumption: Vmax represents the maximum velocity, K_m is the substrate concentration at 50% of Vmax. S is the substrate (oxygen) concentration. (d) Mass-balance equation based on Fick's second law: C_a represents oxygen concentration, r oxygen consumption rate, D_o is the diffusion coefficient.

Supplementary Fig. 2. Rheology measurements for the gelatin hydrogel

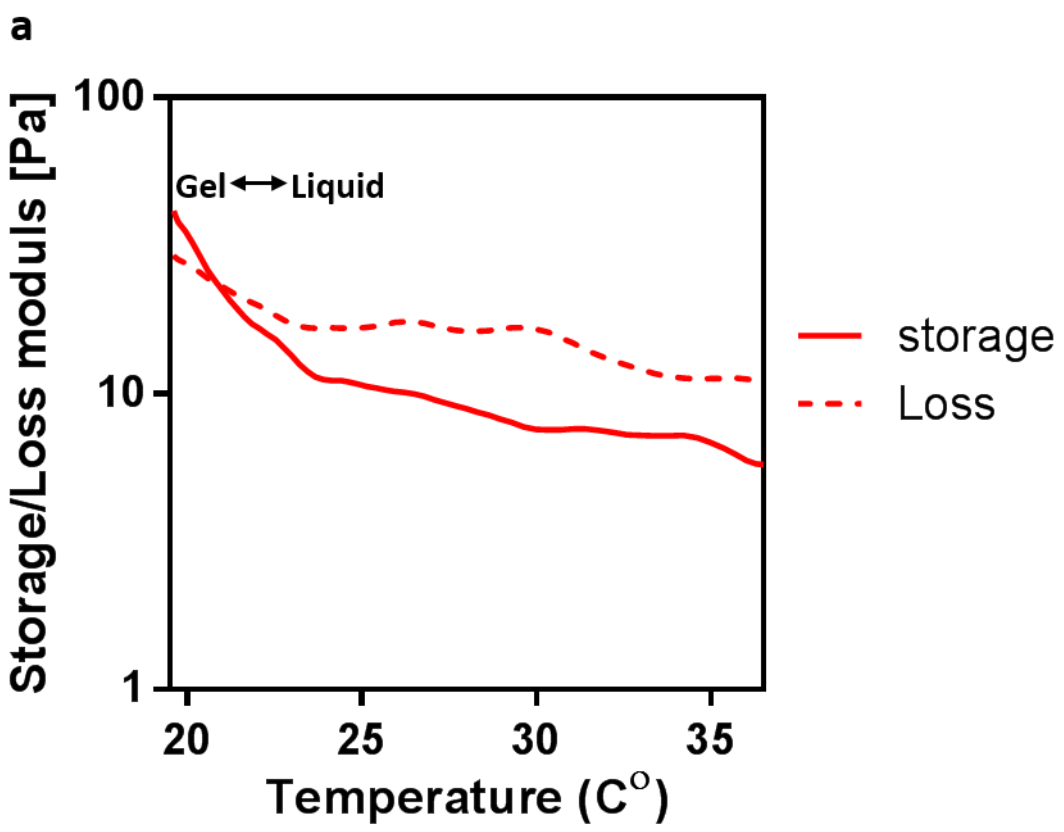

(a) Rheology measurements showing the thermoresponsive quality of the gelatin hydrogel.

Supplementary Fig. 3. Patch perfusion and structural integrity.

a

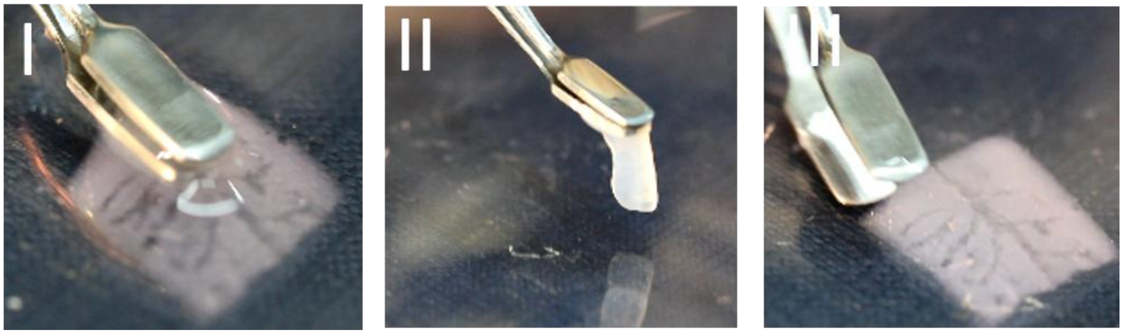

b

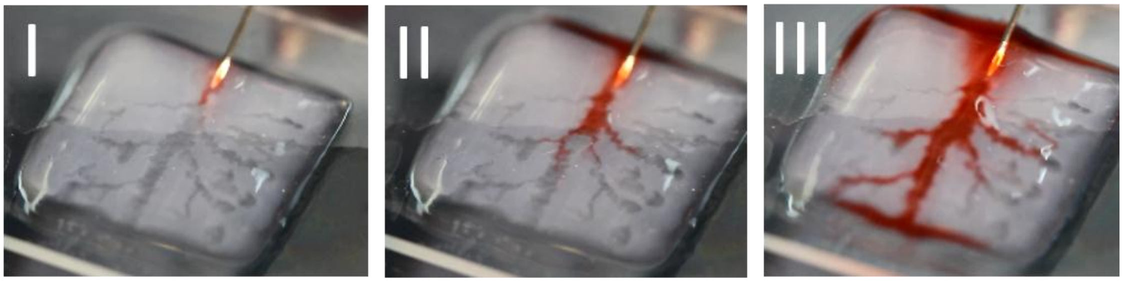

(a) Patch lifting and immersion. (b) Patch perfusion.

Supplementary Fig. 4. Top view of the printed blood vessel.

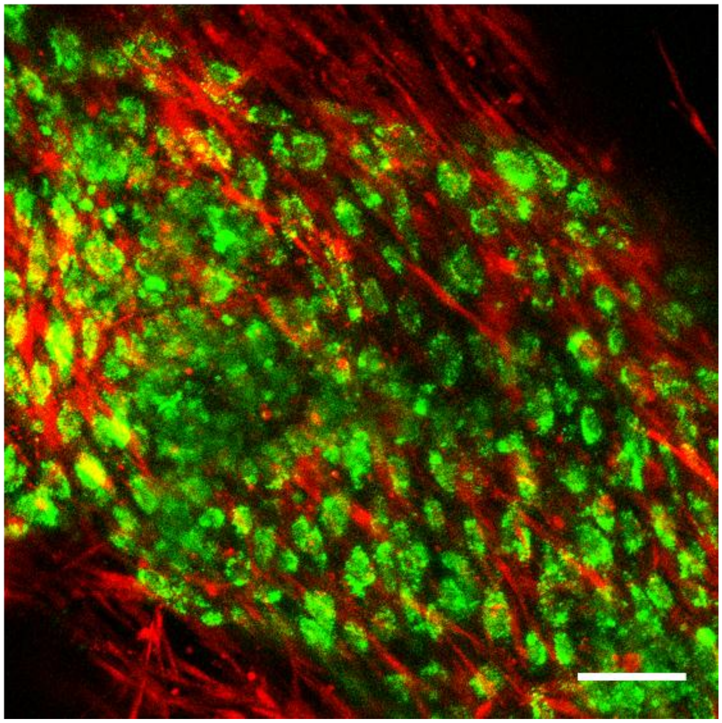

A top view of the printed blood vessel, showing the interactions between the ECs (green) and the HNDFs (red). Scale bar= 100 µm.

Supplementary Fig. 5. Cross section of the printed explant.

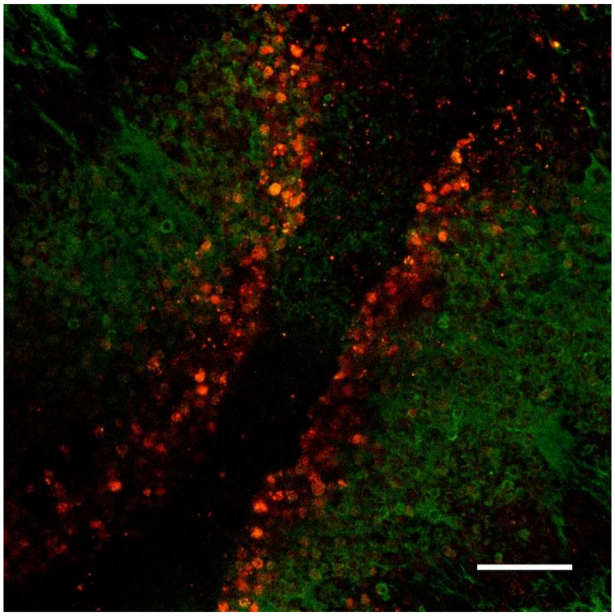

Cross section of the printed explant immunostained against sarcomeric actinin (green). ECs express RFP (red). Scale bar= 100 µm.

Supplementary Fig. 6. The support medium microparticulate material

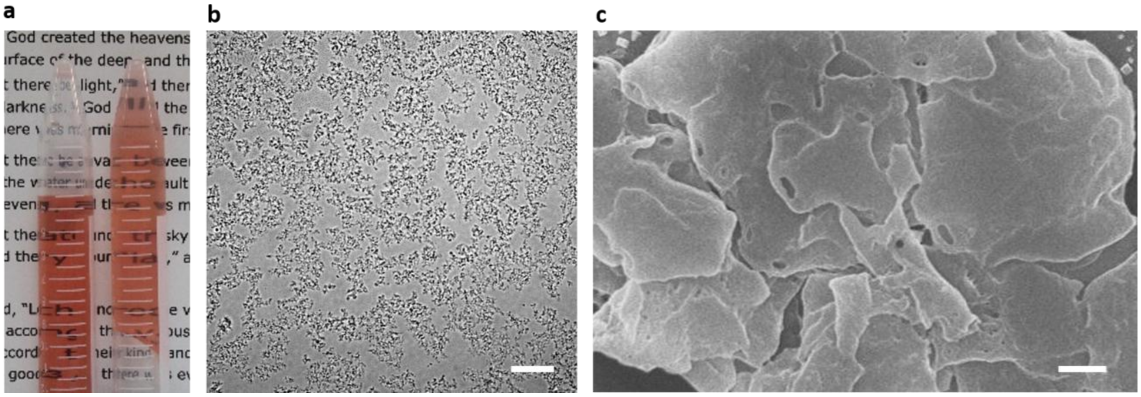

(a) An image showing the transparency of culture medium (left) and the support medium (right). (b) Light microscopy and (c) SEM image of the microparticulate material. Scale bars: b= 50 µm, c= 1 µm.

Supplementary Fig. 7. A crisscross structure, printed within the support medium.

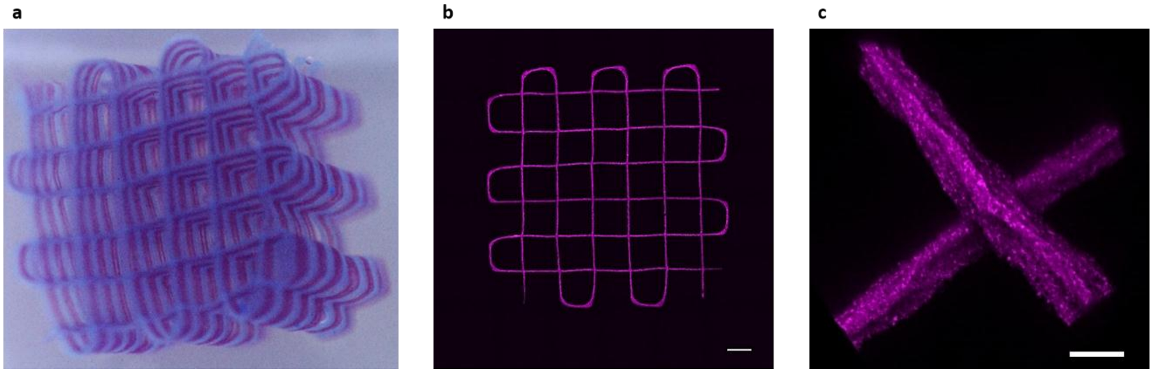

(a) A thick, multi-layered crisscross structure (two colored; red and blue) after extraction from the support medium. (b) A confocal image of a two-layer crisscross structure printed with the personalized hydrogel, and (c) The intersection of two strands. Scale bars: (b)=1 mm, (c)= 100 µm.

Supplementary Fig. 8. Printing scheme of a representative part of the thick lumen-containing consturct.

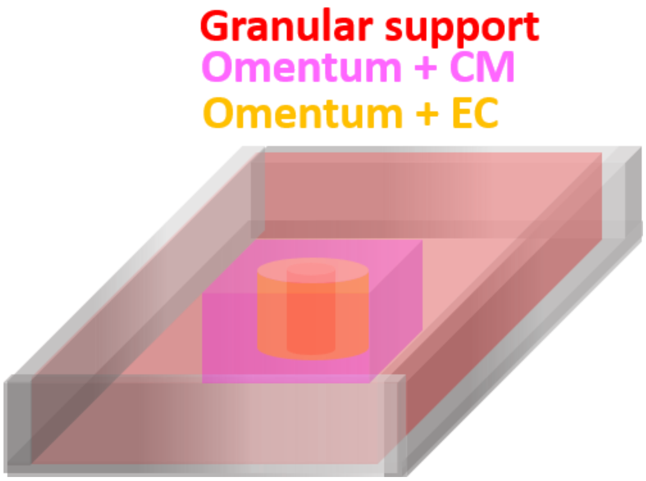

Supplementary Fig. 9. The printed heart.

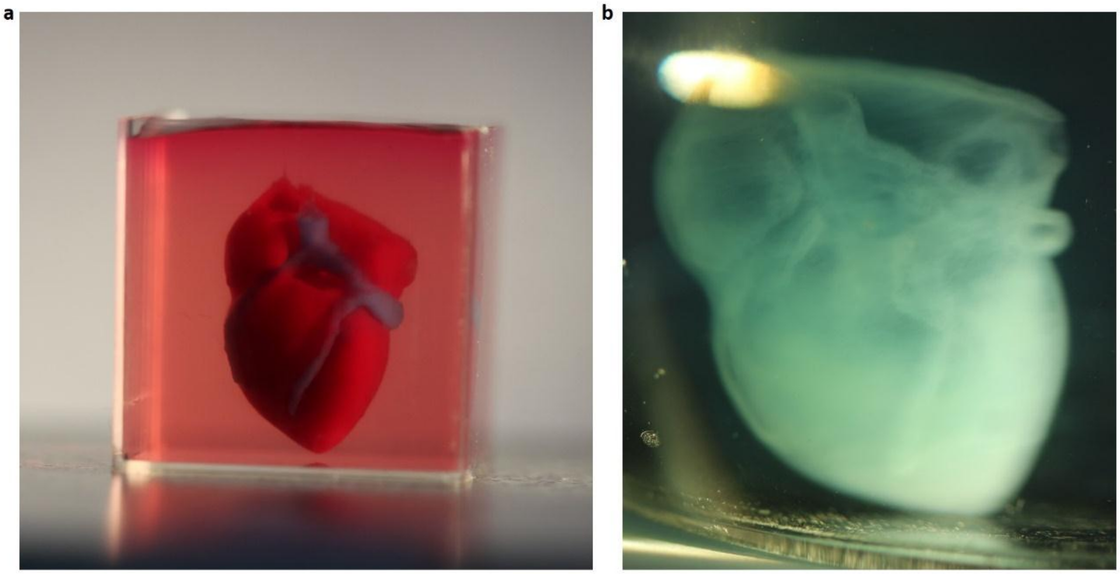

(a) The printed heart in the support medium at the end of the printing process. (b) An extracted heart in PBS.

Supplementary Fig. 10. Compression test of printed hearts.

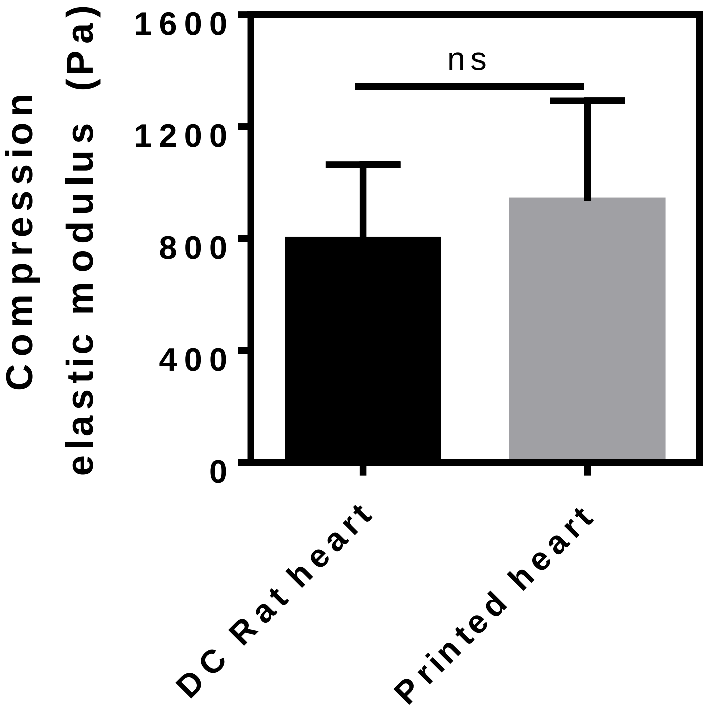

Supplementary Fig. 11. Human heart model printing program.

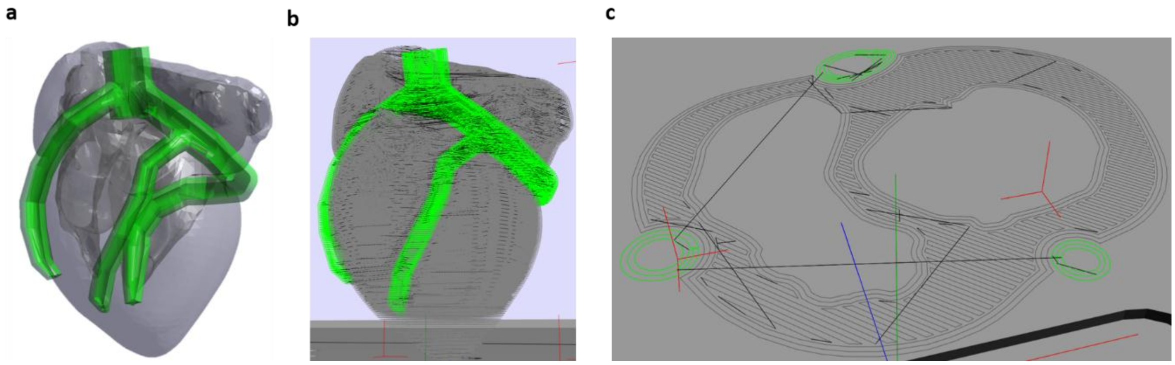

(a) The heart CAD model. (b) The CAD model when sliced by the printing program into layers. (c) One layer of the printing program. (grey – CM bioink, green – EC bioink).

Supplementary movies

Supplementary Movie 1.
3D printing of patient-specific vascularized cardiac patch.

Supplementary Movie 2.
Lift and resuspension of the vascularized patch.

Supplementary Movie 3.
Infusion of red dye into the open lumens of the vascularized cardiac patch.

Supplementary Movie 4.
3D confocal image of a GFP HUVEC (green) and RFP HNDF (red) lumen.

Supplementary Movie 5.
Calcium imaging of a printed vascularized cardiac patch. Scale bar = 100 µm.

Supplementary Movie 6.
Calcium imaging of a printed cardiac patch wide view. Scale bar = 500 µm.

Supplementary Movie 7.
Calcium imaging of a printed cardiac patch near view. Scale bar = 100 µm.

Supplementary Movie 8.
Confocal 3D image of an immunostained section of the cardiac patch, extracted 7 days post transplantation, actinin (red), nuclei (blue).

Supplementary Movie 9.
Printing a double crisscross in a support bath.

Supplementary Movie 10.
Printing concentric spheres in a support bath.

Supplementary Movie 11.
Printing of a human hand.

Supplementary Movie 12.
Confocal image of a lumen entry.

Supplementary Movie 13.
Tripod perfusion.

Supplementary Movie 14.
Printing a heart in a support bath.

Supplementary Movie 15.
Manipulation of the printed heart in PBS after extraction.

Supplementary Movie 16.
Perfusion through the printed heart.

Supplementary Movie 17.
A printed cellularized heart, CM (pink), EC (orange).

Supplementary Movie 18.
Confocal image of printed heart compartments.
